# The *Clostridium* Metabolite P-Cresol Sulfate Relieves Inflammation of Primary Biliary Cholangitis by Regulating Kupffer Cells

**DOI:** 10.3390/cells11233782

**Published:** 2022-11-26

**Authors:** Hai-Yan Fu, Jia-Min Xu, Xin Ai, Fu-Tao Dang, Xu Tan, Hai-Yan Yu, Juan Feng, Wen-Xia Yang, Hai-Tao Ma, Rong-Fang Tu, Ajay Kumar Gupta, Lagan Kumar Manandhar, Wei-Min Bao, Ying-Mei Tang

**Affiliations:** 1 Gastroenterology Department the Second Affiliated Hospital of Kunming Medical University, No. 374 Dianmian Avenue Wuhua Area, Kunming 650101, China; 2 Hepatobiliary Surgery Department First People’s Hospital of Yunnan, No. 157 Jinbi Road Xishan Area, Kunming 650101, China

**Keywords:** primary biliary cholangitis, p-Cresol sulfate, Kupffer cells, inflammation

## Abstract

Objective: To study the effect and mechanism of the *Clostridium* metabolite p-Cresol sulfate (PCS) in primary biliary cholangitis (PBC). Methods: Gas chromatography-mass spectrometry (GC-MS) was used to detect differences in tyrosine, phenylalanine, tryptophan, PCS, and p-Cresyl glucuronide (PCG) between the serum of PBC patients and healthy controls. In vivo experiments, mice were divided into the normal control, PBC group, and PBC tyrosine group. GC-MS was used to detect PCS and PCG. Serum and liver inflammatory factors were compared between groups along with the polarization of liver Kupffer cells. Additionally, PCS was cultured with normal bile duct epithelial cells and Kupffer cells, respectively. PCS-stimulated Kupffer cells were co-cultured with lipopolysaccharide-injured bile duct epithelial cells to detect changes in inflammatory factors. Results: Levels of tyrosine and phenylalanine were increased, but PCS level was reduced in PBC patients, with PCG showing a lower concentration distribution in both groups. PCS in PBC mice was also lower than those in normal control mice. After oral administration of tyrosine feed to PBC mice, PCS increased, liver inflammatory factors were decreased, and anti-inflammatory factors were increased. Furthermore, Kupffer cells in the liver polarized form M1 transitioned to M2. PCS can damage normal bile duct epithelial cells and suppress the immune response of Kupffer cells. But PCS protects bile duct epithelial cells damaged by LPS through Kupffer cells. Conclusions: PCS produced by *Clostridium*-metabolized tyrosine reduced PBC inflammation, suggesting that intervention by food, or supplementation with PCS might represent an effective clinical strategy for treating PBC.

## 1. Background

Primary biliary cholangitis (PBC) is an immune-mediated disease characterized by the destruction of intrahepatic bile ducts at the beginning of the disease, followed by progressive damage to the bile duct tree, cholestasis, and eventually liver fibrosis and even liver cancer [1,2,3]. The incidence of PBC in women is significantly higher than that in men, and PBC pathogenesis remains uncertain but involves interactions between genetic, immune, and environmental factors [4]. Regrettably, there is no cure for PBC, and given the high recurrence rate following transplantation and the limited source of donors, liver transplantation is also not an optimal choice [3].

PBC-related intestinal flora changes, including reduced levels of protective metabolites and increased levels of damaging metabolites [5]. p-Cresol sulfate (PCS) and p-Cresyl glucuronide (PCG) are metabolites of *Clostridium*. Food and endogenous proteins and peptides are decomposed by digestive enzymes to form aromatic amino acids, such as tyrosine and phenylalanine, which are fermented by *Clostridium*, metabolized into PCS and PCG in the liver, and finally excreted through the urinary system [6]. PCS has long been recognized as a uremic toxin [7,8], however, there are few studies on the role of PCS in the liver.

As a sentinel of the liver-specific immune system, Kupffer cells are responsible for processing invading bacterial metabolites [9]. Our previous studies found that in PBC mice, Kupffer cells are activated and damage bile duct epithelial cells via cytokines and participate in the development of diseases [10]. Current study aimed to explore the effects and mechanisms of the *Clostridium* metabolite PCS in PBC.

## 2. Material and Methods

### 2.1. Inclusion and Exclusion Criteria for Clinical Research Subjects

This study was approved by the Ethics Committee of the Second Affiliated Hospital of Kunming Medical University (review-PJ-2019-38; Chinese clinical trial registration no. ChiCTR2000040564). All patients met the diagnostic criteria for PBC according to the Chinese Society of Liver Diseases, Chinese Medical Association Gastroenterology, and Chinese Medical Association Infectious Diseases in 2015 [11]: (1) increased biochemical indicators of cholestasis; (2) positive for serum anti-mitochondrial antibody or anti-mitochondrial antibody M2 subtype; (3) liver histopathology compatible with PBC. Patients meeting two of these requirements were included in the study. Exclusion criteria were as follows: (1) infectious diseases, other hereditary diseases, except PBC, and/or combination with malignant tumors, mental diseases, or other diseases; (2) long-term use of medication, heavy drinking, and/or drug use; (3) current pregnancy or lactation. Any one of the above conditions resulted in exclusion.

### 2.2. Animals

Specific pathogen-free (SPF) C57BL/6 female mice (4–6 weeks old; weight: 18–22 g) were purchased from the Experimental Animal Research Center of Kunming Medical University and raised in the SPF Laboratory of the Experimental Animal Research Center of Kunming Medical University. The experimental procedure complied with regulations on the management of experimental animals at Kunming Medical University, and the mouse sacrifice process complied with the ARRIVE guidelines. The 20 mice in the pre-experimental group were divided into a tyrosine feed group (See Supplementary Table S1 for the composition of tyrosine feed) and a normal control group (*n* = 10/group). Mice in the experimental group (*n* = 25) were divided into three groups: the PBC model group (*n* = 7), the normal control group (*n* = 10), and the PBC tyrosine treatment group (*n* = 8). Chemical reagents were used to prepare PBC mice according to previously described methods, with slight modifications [10]. Briefly, PBC mice were intraperitoneally injected with 2-octynoic acid conjugated bovine serum albumin (2OA-BSA, Beijing Hapten and Protein Biomedical Institute, Beijing, China), completing Freund’s adjuvant (Sigma-Aldrich, Saint Louis, MA, USA) once daily for 3 days. Polyinosinic:polycytidylic acid (Poly I:C, Merck Millipore, Billerica, MA, USA) was injected intraperitoneally on days 3 and 6 (5 mg/kg). From weeks 4 through 12, poly I:C (300 μg/mouse) was intraperitoneally injected once every 3 days, and at week 12, the PBC animal model was established. The normal control group was provided standard synthetic mouse feed supplied by the Animal Experiment Center of Kunming Medical University, and at a ratio of 3% fat, 20% protein, and 77% carbohydrate. Tyrosine feed was provided by Jiangxi Synergy Pharmaceutical Co., Ltd. (Nanchang, China), as required, and stored at 4 °C.

### 2.3. Cultivation and Co-Cultivation of Kupffer Cells and Intrahepatic Biliary Epithelial Cells

Liver Kupffer cells and intrahepatic bile duct epithelial cells from Sprague–Dawley rats were purchased from Shenzhen Haodi Huatuo Biotechnology Co., Ltd. (Shenzhen, China). Co-culture was performed using a transwell chamber (Mengzhuang Technology Co., Ltd., Beijing, China), in which the upper chamber comprised bile duct epithelial cells and the lower chamber included Kupffer cells. According to the results of pre-experiments, 100 ng/mL lipopolysaccharide (LPS, Sigma-Aldrich, Saint Louis, MA, USA) was used to damage bile duct epithelial cells over the course of 4 h. The total co-cultivation time was 12 h.

### 2.4. Hematoxylin and Eosin (H&E) Staining

Following sacrifice, livers from mice were immediately fixed in neutral buffered formalin (10%, Solarbio, Beijing, China). After dehydration with an ethanol gradient (Biojet Co., Ltd., Kunming, China), infiltration, embedding, and slicing, sections were stained with H&E and imaged with an inverted optical microscope (CKX53; Olympus, Tokyo, Japan) to assess liver inflammation. Liver damage was evaluated according to a previous Ludwig and Scheuer histologic classification method [12].

### 2.5. Gas Chromatography Mass Spectrometry (GC-MS) Measurement of Aromatic Amino Acids and PCS/PCG

Serum was collected from patients, and serum, liver tissue, and cecum contents collected from mice were homogenized individually. Serum (20 µL) was mixed with 380 mL of acetonitrile/water extract (Sigma-Aldrich, Saint Louis, MA, USA) containing 5 µM chlorpropamide. Frozen liver sections (100 mg) and cecum contents (10 mg) were separately ground and mixed with 500 µL of acetonitrile/water (Merck & Co., Inc., Rahway, NJ, USA) (containing the internal standard chlorpropamide 5 µM), respectively. The mixtures were shaken at room temperature for 20 min, followed by centrifugation at 4 °C and 15,000 rpm for 20 min. The supernatant (100 µL) was diluted with 100 µL of pure acetonitrile and mixed thoroughly. Serum, liver tissue, and cecum contents were all sonicated for 5 min, centrifuged at 18,000× *g* at 4 °C for 20 min, and the supernatant was removed for GC-MS analysis (Agilent, Palo Alto, CA, USA), as previously described [13]. Authentic standards of PCS/PCG were obtained from APExBIO (Boston, MA, USA). Ultra-performance liquid chromatography time-of-flight MS was performed using a Dionex Ultimate 3000 (Thermo Fisher Scientific, Waltham, MA, USA).

### 2.6. Quantitative Real-Time Polymerase Chain Reaction (PCR)

Total RNA from treated cells and frozen liver was extracted using Trizol reagent (Life Technologies, Carlsbad, CA, USA). RNA was treated with 0.2 mL chloroform (Solarbio, Beijing, China) and 0.4 mL isopropanol (Solarbio, Beijing, China), followed centrifugation at 20 °C. The pellet was resuspended in 0.5 mL diethyl pyrocarbonate-treated water (Solarbio, Beijing, China), and 2 µL was used to determine concentration. PCR was performed according to manufacturer instructions for the reverse transcription kit (Tiangen Biotechnology Co., Ltd., Beijing, China). RNA was added to a mixture of 5× *g* DNA buffer (Aidlab Biotechnologies Co., Ltd., Beijing, China), FQ-RT primer mix (Thermo Fisher Scientific, Waltham, MA, USA) 2 μL, 10× King RT buffer (Tiangen Biotechnology Co., Ltd., Beijing, China), and RNase-free ddH_2_O (Tiangen Biotechnology Co., Ltd., Beijing, China) to a final volume of 10 μL and incubated at room temperature. PCR cycling conditions were as follows: 42 °C for 15 min and 95 °C for 3 min, followed by 1 cycle of 30 °C for 10 min, 42 °C for 1 h, 95 °C for 5 min, and a final rest period at 12 °C. Primers are listed in Supplementary Table S2.

### 2.7. Western Blot Analysis

Total protein was extracted with RIPA (radio immunoprecipitation assay) lysate. The protein concentration was determined using a BCA protein assay kit (Thermo Fisher Scientific, Waltham, MA, USA) 50 μg of protein samples were subjected to SDS-PAGE and transferred onto PVDF membranes (Solarbio, Beijing, China). The membrane was blocked with 5% nonfat dry milk (Solarbio, Beijing, China) for 1.5 h, then incubated with specific primary antibodies at 4 °C overnight. Following incubation with HRP-conjugated secondary anti-rabbit antibody (7074, Cell Signaling Technology, Danvers, MA, USA), membranes were examined with ECL reagent (KF005, Affinity Biosciences, Melbourne, Australia) and visualized on ChemiDocTM XRS + Imaging System (BioRad, Hercules, CA, USA). ImageJ software (U.S. National Institute of Health, Bethesda, MD, USA), was applied to detect and analyze the gray values of protein bands on the membrane. The following primary antibodies: transforming growth factor (TGF)-β, interleukin (IL)-10, tumor necrosis factor (TNF)-α, interleukin(IL)-6, NADPH oxidase 2 (NOX2), P22, arginase-1 (Arg-1), C–C motif chemokine ligand 3 (CCL3), interleukin(IL)-1β, monocyte chemoattractant protein-1 (MCP-1), C–X3–C motif chemokine ligand 1 (CX3CL1), and β-actin. All the above antibodies purchased form Proteintech, Beijing, China.

### 2.8. Enzyme-Linked Immunosorbent Assay (ELISA)

The serum of mice and culture supernatant was collected. The levels of IL-1β, IL-6, IL-8, IL-10, TGF-β, TNF-α, CCL3, CX3CL1, and MCP-1 were measured by ELISA Kit (Jianglai Biotechnology, Shanghai, China) according to manufacturer instructions.

### 2.9. MS Analysis

MS data were processed using Mass Profinder (Agilent Technologies, Santa Clara, CA, USA) in order to generate a data matrix that included the sample name, retention time, mass-to-charge ratio, and ion-peak area. To improve data quality and reliability, the matrix was generated using quality control samples, and variables with a coefficient of variation >30% were filtered out. Subsequently, SIMCA-P + 13.0 (Umetrics, Umeå, Sweden) was used to perform multivariate statistical analysis on the dataset, including principal component analysis and orthogonal partial least squares discriminant analysis (OPLS-DA). Structures of candidate compounds were matched and determined through secondary fragments and standard products corresponding to the different compounds.

### 2.10. Statistical Analysis

Data fitting a normal distribution are expressed as the mean ± standard deviation, with a two-tailed Student’s *t*-test used to determine differences between groups, and comparisons between multiple groups were performed using analysis of variance or a rank sum test. Correlation coefficients were calculated using Spearman correlation analysis. Statistical analyses and data visualization were performed using OriginPro software (v.2018; OriginLab, Northampton, MA, USA), SPSS software (v.22.0; IBM Corp., Armonk, NY, USA), and GraphPad Prism software (v.6.0; GraphPad Software, San Diego, CA, USA). Photoshop (v.2018; Adobe, Mountain View, CA, USA) was used to process images. *p* < 0.05 was considered significant.

## 3. Results

### 3.1. Unbalanced Aromatic Amino Acids and Metabolic Perturbation in PBC Patients

**Figure 1:**
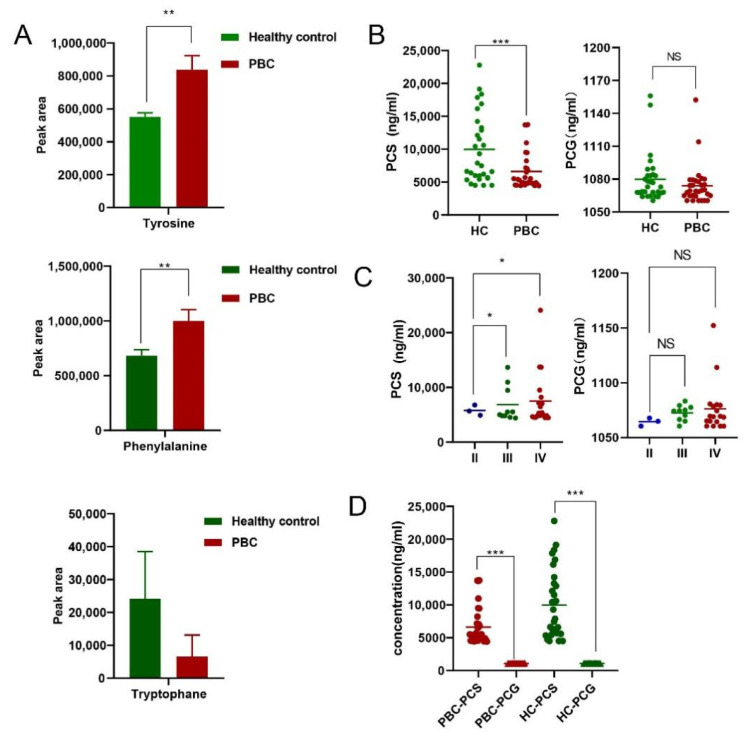
Aromatic amino acids and their metabolite end products PCS/PCG. (**A**) Differences in the peak areas of tyrosine, phenylalanine, and tryptophan between groups. (**B**) Concentration distribution of PCS and PCG between groups. (**C**) Distribution of PCS and PCG at different clinical stages of PBC. (**D**) Concentration distribution of PCS and PCG between groups. * *p* < 0.05, ** *p* < 0.01, and *** *p* < 0.001. NS: no significance.

Levels of tyrosine and phenylalanine differed between PBC patients and healthy controls. Tryptophan abundance value was detected in one patient with PBC and three healthy controls, the statistical analysis was not carried out (Figure 1A). Interestingly, as the end product of tyrosine metabolism, PCS level was significantly reduced in PBC patients, and PCG showed a lower concentration distribution in both groups (Figure 1B). Additionally, PCS concentration in PBC stage II patients was lower than that in stage III and IV patients, whereas no difference was observed in PCG levels (Figure 1C). In both the PBC group and healthy controls, PCS and not PCG was mainly distributed in the serum (Figure 1D).

### 3.2. The Effect of Food-Derived PCS on Normal C57BL/6 Female Mice

**Figure 2:**
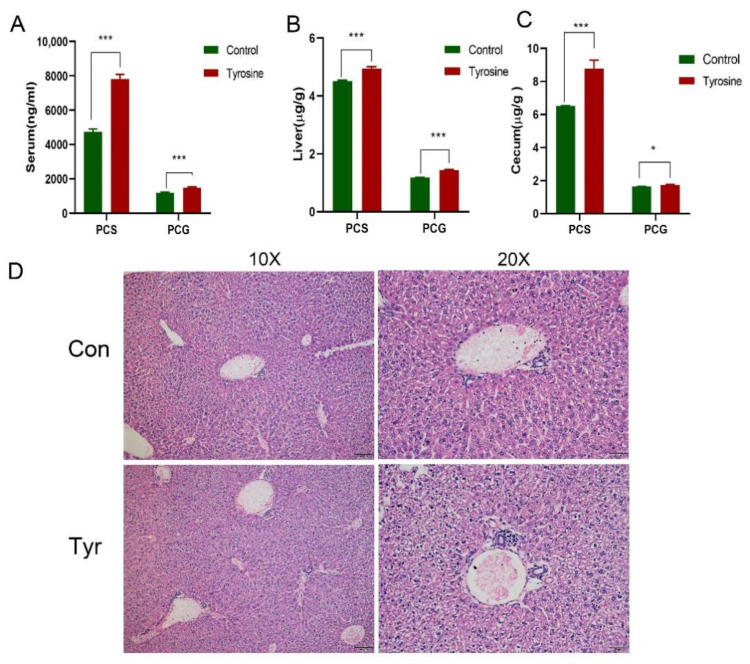
Tyrosine supplementation increases PCS and PCG concentrations. Increases in PCS and PCG concentrations in (**A**) serum, (**B**) liver, and (**C**) cecum. (**D**) PCS administration damages the liver of normal C57BL/6 female mice (*n* = 10). * *p* < 0.05, *** *p* < 0.001.

To increase the concentration of PCS in mice, C57BL/6 female mice were fed a 5% tyrosine diet [14], followed by the detection of PCS and PCG concentrations in the liver, serum, and cecum, respectively. After 10 days, concentrations of PCS and PCG in the serum (Figure 2A), liver (Figure 2B), and cecum (Figure 2C) increased, with higher concentrations of PCS observed. Lymphocytes infiltration in the portal and biliary ducts was significantly reduced in treated mice compared with PBC mice. Additionally, we found that food-derived PCS caused liver damage in normal mice. Specifically, the structure of the liver lumen was complete and clear, increased in hepatocytes volume, the cytoplasm was loose and reticular, and some hepatocytes were translucent, but inflammation was not obvious (Figure 2D); Since tyrosine can also be metabolized to produce phenol, we also tested the concentration of phenol, however, phenol concentrations in mouse liver and serum were lower than the detection limit. Therefore, we speculate that the final product of tyrosine food metabolism was mainly PCS, and the physiological changes in the body should also be caused by PCS changes.

### 3.3. The Effect of Food-Derived PCS on PBC Mice

**Figure 3:**
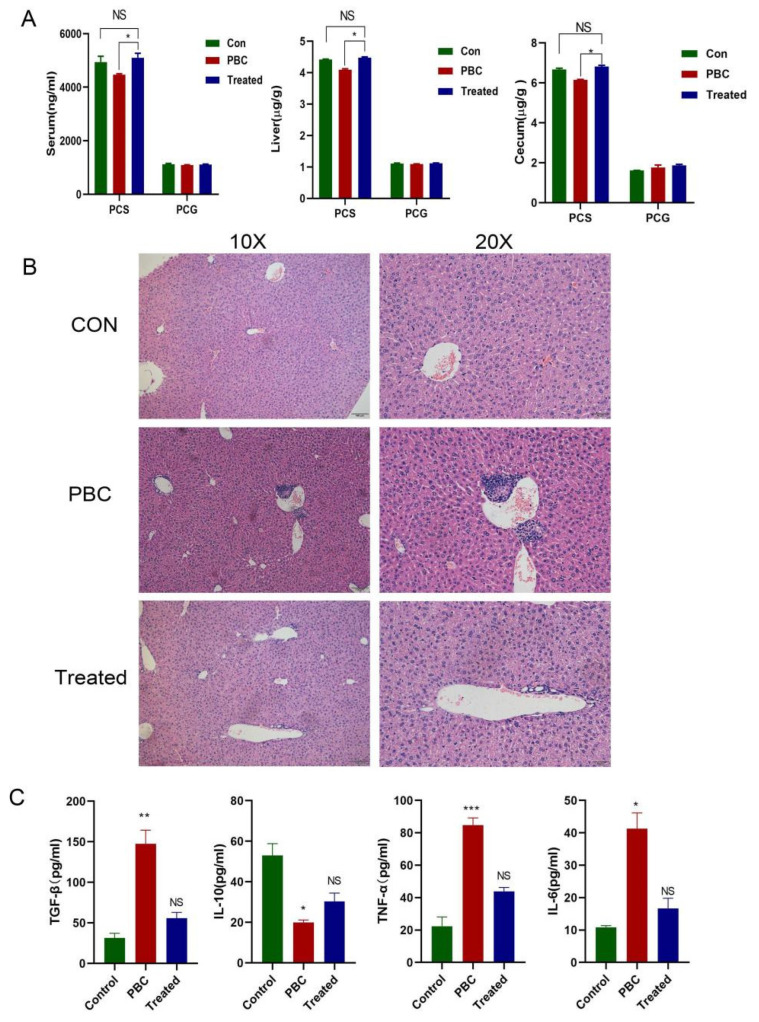
The effect of food-derived PCS on PBC mice. (**A**) A tyrosine-rich diet increased PCS concentrations in serum, liver, and cecum in PBC mice. (**B**) Liver inflammation in PBC mice was partially reduced by food-derived PCS. (**C**) Level of IL-6, IL-10, TNF-α, and TGF-β in serum from each group. PBC model group (*n* = 7), normal control group (*n* = 10), and PBC tyrosine treatment group (*n* = 8). * *p* < 0.05, ** *p* < 0.01, and *** *p* < 0.001. NS: no significance.

We validated the PBC mouse model successfully according to the method described previously [10]. Cholangitis and granulomas were observed in the liver of PBC mice (Figure 3B). A decrease in PCS level in mouse serum, liver, and cecum was identified, which was consistent with findings in serum from PBC patients; however, PCG levels did not change significantly, with PCS concentrations consistently higher than those of PCG. PBC mice fed a tyrosine-rich diet for 10 days showed elevated PCS levels, with serum levels close to those in control mice (Figure 3A). Interestingly, lymphocytes infiltration in the portal and biliary ducts was significantly reduced in treated mice compared with PBC mice, but there are no obvious morphological changes in intrahepatic bile duct epithelial cells (Figure 3B). Additionally, ELISA results showed that IL-6, TNF-α, and TGF-β levels in serum from PBC mice increased along with decreased IL-10 levels relative to controls, whereas these findings were reversed in the PCS-treated group (IL-6, TNF-α, and TGF-β levels decreased, and IL-10 level increased), which showed levels similar to the control group (Figure 3C).

**Figure 4:**
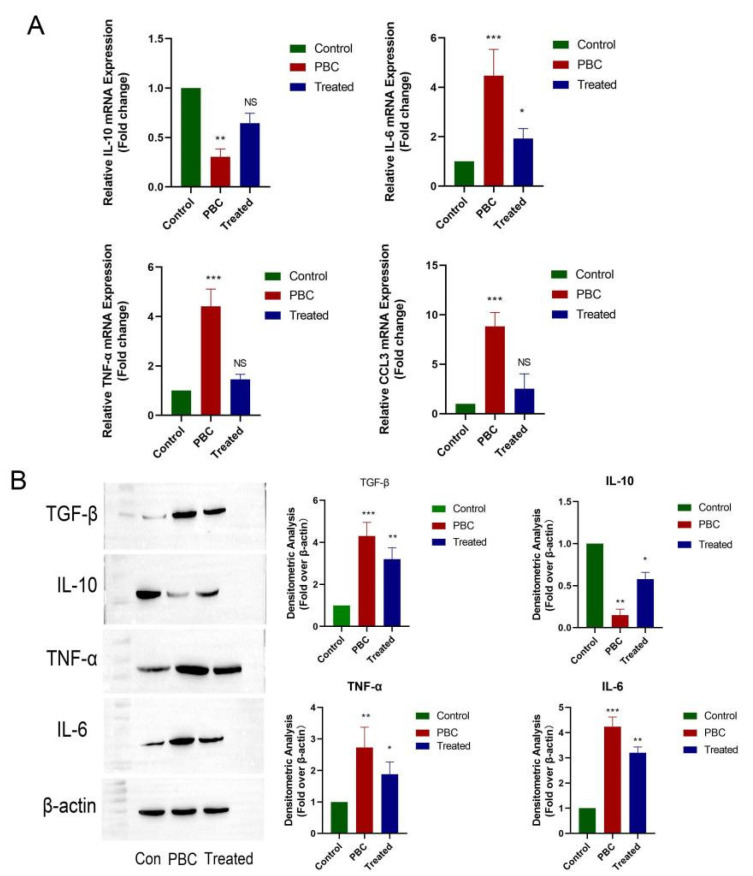
Changes in inflammatory cytokine levels in vivo. (**A**) mRNA levels of IL-6, TNF-α, IL-10, and CCL3. (**B**) Protein levels of TGF-β, IL-10, TNF-α, and IL-6 relative to controls (*n* = 3/group). * *p* < 0.05, ** *p* < 0.01, and *** *p* < 0.001. NS: no significance.

To confirm the reversal of liver inflammation in PCS-treated PBC mice, we detected mRNA levels of IL-6, TNF-α, IL-10, and CCL3 by PCR in liver tissue from each group of mice (Figure 4A), followed by confirmation of protein levels by western blot (Figure 4B). The results showed that both mRNA and protein levels of inflammatory cytokines were elevated in PBC mice along with decreased levels of anti-inflammatory cytokines, whereas the opposite trend was observed in PCS-treated mice, which was consistent with ELISA results.

### 3.4. Effect of Food-Derived PCS on Polarization of Mouse Liver Kupffer Cells

We investigated Kupffer cell activation in the liver of each group to determine the effect of PCS on Kupffer cell polarization. The results showed a significantly higher number of CD86 cells (M1 Kupffer cells) in PBC mice relative to controls (Supplementary Figure S1A), whereas the number of CD206 cells (M2 Kupffer cells) in the PCS-treated group increased significantly relative to control mice (Supplementary Figure S1B), with concordant changes in the M1:M2 ratio observed in each group (Supplementary Figure S1C).

### 3.5. The Effect of PCS on Bile Duct Epithelial Cells In Vitro

Bile duct epithelial cells are targeted for destruction during PBC, however, the effect of PCS on normal bile duct epithelial cells remains unclear. PCS is involved in regulating inflammation in fat cells, endothelial cells, proximal tubular cells, and glial cells [15,16,17,18] while also being responsible for damaging vascular endothelial cells and smooth muscle cells, mainly by elevating oxidative stress [19]. PCS was added to the culture of normal bile duct epithelial cells for various time periods (0, 1, 2, 3, and 4 h), with the highest fluorescence intensity of released reactive oxygen species (ROS) observed at 2 h (Supplementary Figure S2A). This increase was suppressed in the presence of transporter protein inhibitor probenecid, wortmannin, and N-acetyl-L-cysteine (NAC) (Supplementary Figure S2B). Probenecid can decrease the levels of NOX2 and P22 (Supplementary Figure S2C). A similar experiment using the different PCS concentrations revealed altered levels of ROS-related molecules according to western blot analyses (Supplementary Figure S2D).

### 3.6. The Effect of PCS on Kupffer Cells In Vitro

**Figure 5:**
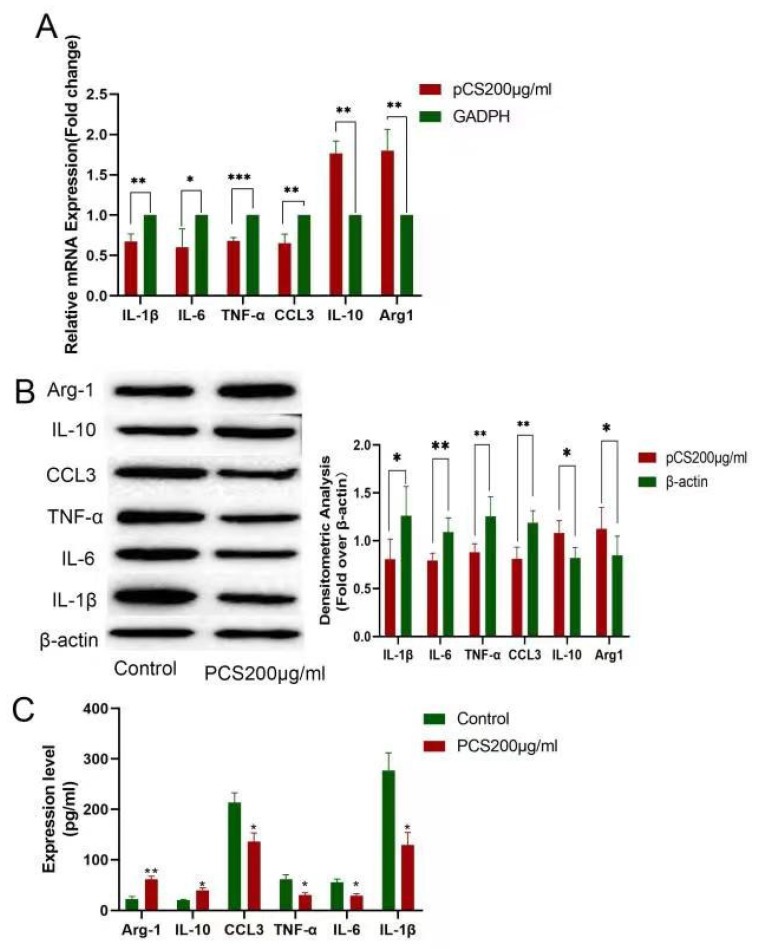
The effect of PCS on Kupffer cells in vitro. (**A**) mRNA and (**B**) protein levels of IL-1β, IL-6, TNF-α, CCL3, IL-10, and Arg-1. (**C**) ELISA confirmation of the same levels in culture supernatant. PBC model group (*n* = 7), normal control group (*n* = 10), and PBC tyrosine treatment group (*n* = 8). * *p* < 0.05, ** *p* < 0.01, and *** *p* < 0.001.

Kupffer cells are the first line of defense for liver immunity; however, the role of PCS on Kupffer cells remains unclear. PCS (200 μg/mL) supplementation to a culture of Kupffer cells for 8 h resulted in decreased mRNA levels of inflammatory cytokines (IL-1β, IL-6. TNF-α, CCL3) and elevated levels of anti-inflammatory cytokines (IL-10 and Arg-1) (Figure 5A), with similar changes in protein levels according to the western blot (Figure 5B). These results were subsequently confirmed by ELISA (Figure 5C).

### 3.7. PCS Protects Bile Duct Epithelial Cells Damaged by LPS through Kupffer Cells

Bile duct epithelial cells are damaged during PBC, at which time LPS levels are increased; therefore, LPS is commonly used to damage bile duct epithelial cells in vitro. To determine whether PCS can protect damaged bile duct epithelial cells via Kupffer cells, we administered LPS to damage bile duct epithelial cells, followed by their co-culture with PCS-stimulated Kupffer cells. Co-cultures were divided into four groups: bile duct epithelial cells cultured alone (BEC); 100 ng/mL LPS administered to bile duct epithelial cells and cultured for 4 h (BEC + LPS); co-culture of Kupffer cells with LPS-injured bile duct epithelial cells for 12 h KC + (BEC + LPS); and Kupffer cells treated with 200 μg/mL PCS prior to co-culture with LPS-injured bile duct epithelial cells for 12 h (PCS + KC) + (BEC + LPS).

**Figure 6:**
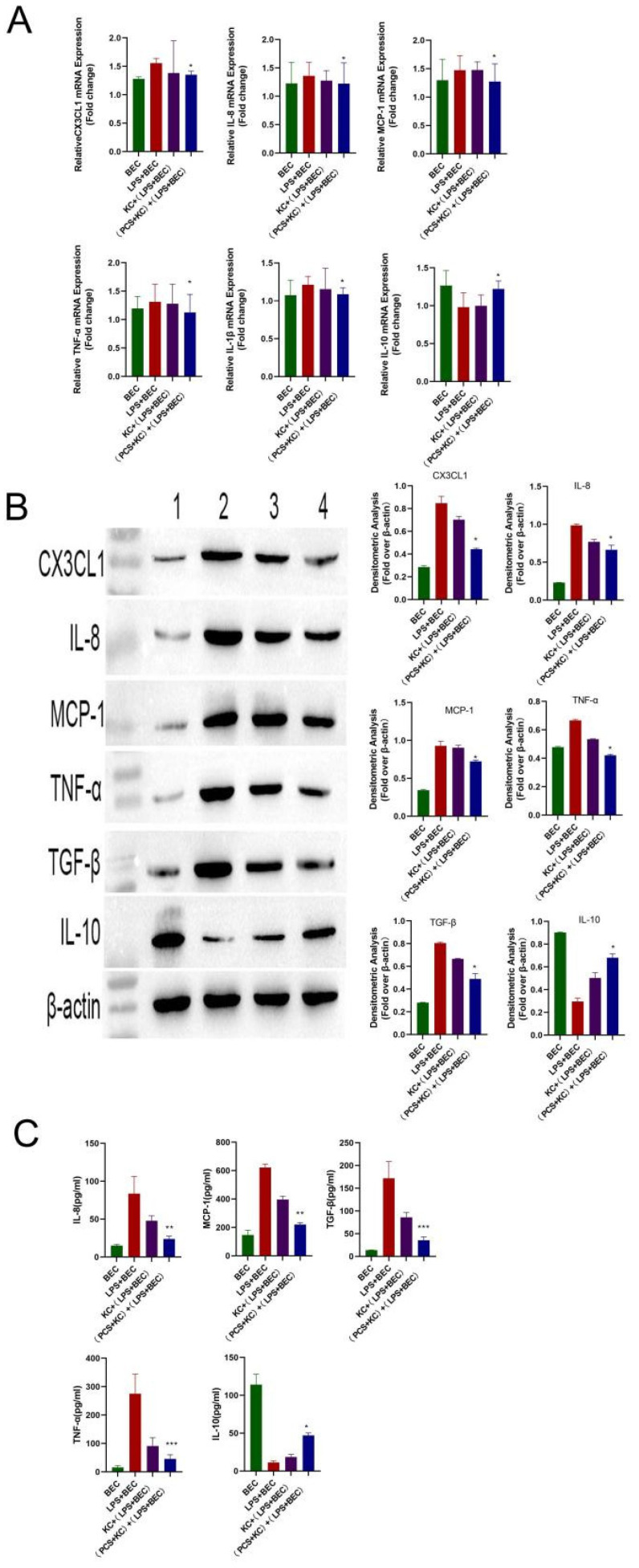
PCS protects LPS-injured bile duct epithelial cells via Kupffer cells. (**A**) mRNA and (**B**) protein levels of CX3CL1, IL-8, MCP-1, TNF-α, IL-1β, and IL-10. (**C**) IL-8, MCP-1, TNF-α, IL-1β, and IL-10 levels measured by ELISA in co-culture supernatant. * *p* < 0.05, ** *p* < 0.01, and *** *p* < 0.001 vs. LPS + BEC group. 1: BEC group; 2: LPS + BEC group; 3: KC + (LPS + BEC) group; and 4: (PCS + KC) + (LPS + BEC) group (*n* = 3/group). KC, Kupffer cells; BEC, bile duct epithelial cells.

**Figure 7:**
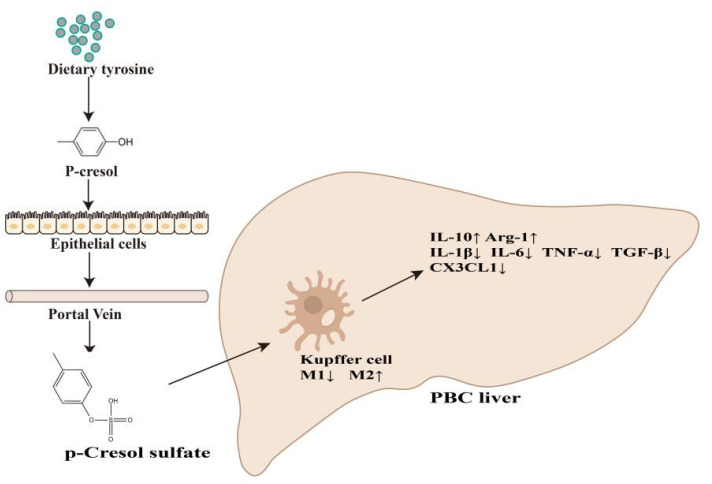
Potential mechanism of PCS-mediated alleviation of PBC inflammation. PCS reduces levels of proinflammatory factors (IL-1β, IL-6, TNF-α, TGF-β, and CX3CL1), increases the level of the anti-inflammatory factor IL-10, Arg-1, and alleviates PBC-related inflammation by changing the polarization of Kupffer cells.

Following LPS stimulation, we observed elevated mRNA levels of inflammatory cytokines (TNF-α, IL-8, MCP-1, IL-1β, and CX3CL1) and decreased mRNA levels of the anti-inflammatory cytokine IL-10 relative to levels observed in the BEC group and consistent with previous results and a clinical PBC phenotype. However, in the (PCS + KC) + (BEC + LPS) group, both mRNA and protein levels of these cytokines recovered to those observed in the BEC group (Figure 6A,B). Kupffer cells without PCS also have the effect of reducing inflammation, but the effect was expanded by PCS. These results were subsequently confirmed by ELISA (Figure 6C), suggesting that PCS-stimulated Kupffer cells protected LPS-damaged bile duct epithelial cells. Altogether, these findings indicated that PCS could reduce PBC-related inflammation by changing the polarization of Kupffer cells (Figure 7).

## 4. Discussion

### 4.1. PCS Can Relieve Inflammation of PBC In Vitro and In Vivo

The world’s first case of PBC was reported in 1851 [20], and increased awareness of the disease has resulted in annual increases in PBC incidence [21]. Currently, the only drugs available for clinical treatment are ursodeoxycholic acid and cholic acid, but about 30% of patients do not respond to these drugs [22]. In the present study, we found that PCS downregulated levels of inflammatory factors in vitro and in vivo. The compounds produced by bacterial metabolism can protect the damaged liver, with supplementation of *Tripterygium wilfordii* propionate a reportedly effective strategy to ameliorate liver toxicity [23]. Additionally, we first found that food-derived PCS damaged normal hepatocytes in mice, we speculated that the toxicity may be caused by accumulated dose. Both indole and PCS are uremic toxins. Indole, produced from dietary tryptophan by bacterial metabolism, strengthens intestinal epithelial barrier integrity [24] to protect against non-alcoholic fatty liver disease [25,26]. Although the anti-inflammatory mechanism of uremic toxins differs, their demonstrated protective effect is clear. A cross-sectional study demonstrated that PBC was associated with altered composition and function of gut microbiota compared with healthy controls, as well as a moderately lower level of diversity. Among those changing genera, the *Clostridium* were significantly increased in PBC, which indicates the *Clostridium* may be involved in the pathogenesis of PBC [27].

### 4.2. PCS Downregulates PBC Inflammation through Kupffer Cells

Kupffer cell heterogeneity and subgroup function are affected by multiple factors, including bile acid, bacteria, and bacterial metabolites [28]. A previous study showed that the bacterial metabolite PCS is present in the circulation and can potentially exert immunomodulatory effects outside the intestine [29]; however, the specific mechanisms underlying immunomodulation by microbiota metabolites in the liver remain poorly understood. Kupffer cells in the liver of PBC patients transform to an M1 phenotype and mainly localize in the portal area, with these activities positively correlated with liver inflammation [30]. In the present study, we showed that in livers from mouse models of PBC, M1 Kupffer cells accounted for the majority of these cells, and that their polarization could be reversed by PCS both in vivo and in vitro. Similarly, a previous study reported that oral administration of indole reduced mouse liver inflammation in a Kupffer-dependent manner, suggesting their significant contribution to the regulation of LPS-induced liver inflammation [31]. LPS levels increase in patients with various etiologies of liver diseases [32,33], and ablation of Kupffer cells or use of probiotics to reduce LPS accumulation has achieved some success in alcoholic liver disease [34].

### 4.3. Targeting PCS Might Be a Promising Strategy for the Treatment of PBC

Due to the rarity of PBC and the lack of readily available licensed therapies, interest has increased in repurposed therapies [35]. PCS is a “micro-inflammatory and low-toxicity” substance that can potentially provide promising effects for PBC treatment. A previous study reported that serum PCS concentrations during renal insufficiency can reach up to 105 mg/L (558.5 μmol/L) [36]. As PCS stimulation cannot be extended indefinitely in vitro, we attempted to administer PCS over the course of time in vivo. After 10 days of oral administration of food-derived PCS to the PBC mouse model, we observed a significant reduction in liver inflammation. Subsequent in vitro experiments showed PCS was beneficial to LPS-damaged bile duct epithelial cells through its alteration of Kupffer cell polarization. However, administration of PCS to normal mice and normal bile duct epithelial cells caused toxicity.

In advanced PBC, liver dysfunction cannot effectively decompose and synthesize nutrients such as protein and amino acids. However, PBC animal models demonstrate the earliest immunological events that occur before the manifestation of the clinical disease [35]. In the present study, the PBC mouse model presented the early stage of PBC, which differed from the phenotype observed in clinically advanced PBC patients. We administered tyrosine feed in order to significantly increase the PCS concentration in the mice, whereas tyrosine levels in patients with advanced PBC patients decrease along with PCS concentration. This trend of increasing substrates and decreasing products suggests the feasibility of tyrosine or PCS supplementation to treat early PBC.

In the current study, we found a decrease in PCS and focused on the effect of PCS on PBC. Whether similar role of PCS in cholestasis, such as PSC, is unknown. Certainly, we will carry out further research to elucidate this issue.

### 4.4. PCS May Be a New Tool for Assisting in PBC Diagnosis

We found that PCS levels differed in the early and late stages of PBC. Decreased PCS levels in the early stage of PBC might be the result of a self-protection mechanism that consumes PCS to fight inflammation. Therefore, PCS reduction might indicate PBC onset. Increased PCS excretion in the urine of Northern Italian centenarians implies that amino acid metabolism and microbial function are key regulatory processes in human aging [37]. Furthermore, elevated PCS levels in drug-induced liver injury represent a possible diagnostic marker of the disease [38]. Additionally, in children with type 1 diabetes, increased serum PCS concentration can be used to monitor the effect of insulin therapy [39]. Although elevated PCS has been used as a diagnostic marker for other conditions, in this study, we found that decreased PCS was indicative of PBC.

## 5. Conclusions

In summary, this study is the first to report that tyrosine-derived PCS improves PBC-related inflammation in vitro and in vivo, with this effect directly related to regulating Kupffer cell polarization. Consequently, intervention via food or supplementation with PCS might represent an effective clinical strategy to treat PBC.

## Data Availability

The data are available from the corresponding author upon request.

## Supplementary Materials

The following supporting information can be downloaded at: https://www.mdpi.com/article/10.3390/cells11233782/s1. Figure S1. Effect of food-derived PCS on polarization of mouse liver Kupffer cells. Figure S2. The effect of PCS on bile duct epithelial cells in vitro. Table S1. Composition of tyrosine feed. Table S2 Primer sequence.
