# Supplementary material for: The Clostridium Metabolite P-Cresol Sulfate Relieves Inflammation of Primary Biliary Cholangitis by Regulating Kupffer Cells
Source: Cells. 2022 Nov 26;11(23):3782. doi: 10.3390/cells11233782 (PMC9736483; doi:10.3390/cells11233782)
Supplement: Supplementary file 1 [file cells-11-03782-s001.zip › cells-1966470-supplementary.pdf]

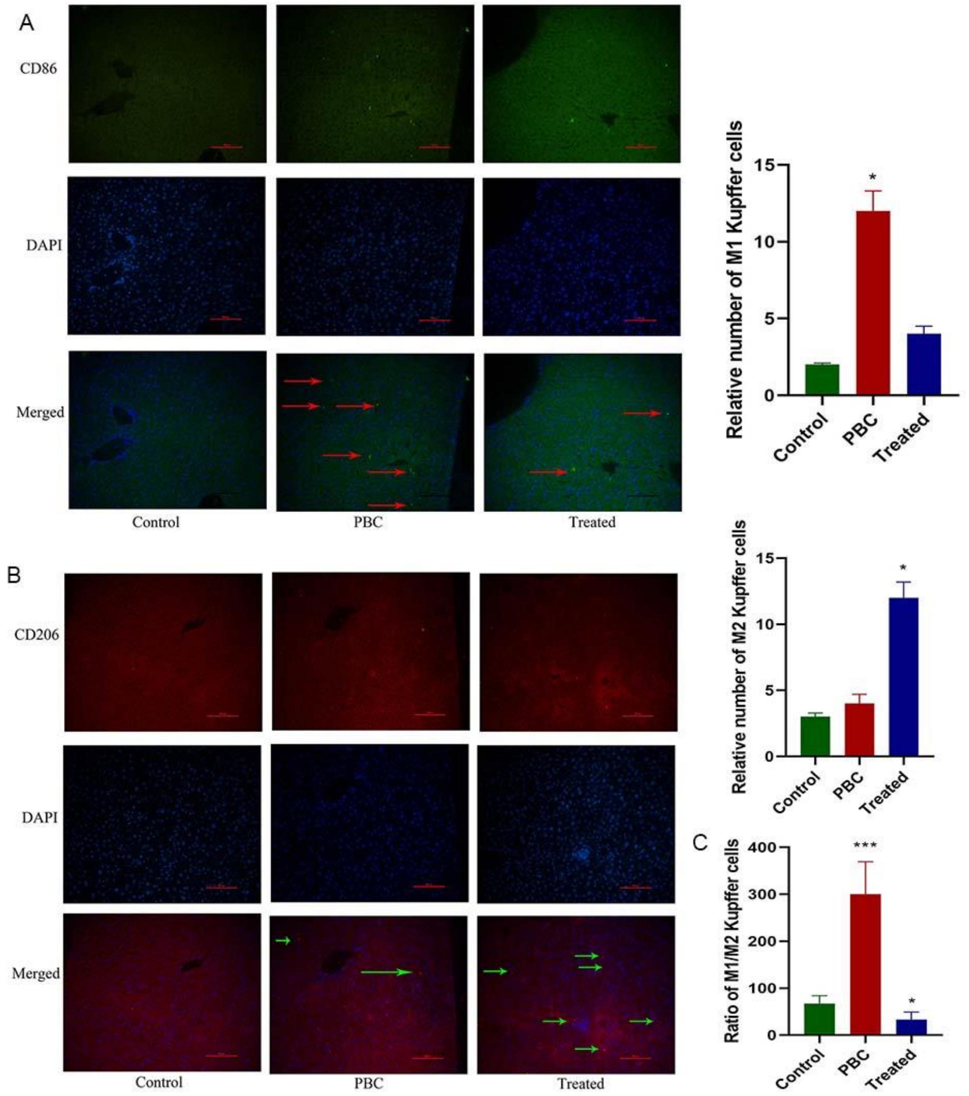

Figure S1. Effect of food-derived PCS on polarization of mouse liver Kupffer cells.

(A) Increases in the number of CD86 Kupffer cells in PBC mice. (B) Increases in the number of CD206 Kupffer cells in PCS-treated mice resulted in a change in the (C) M1:M2 ratio in the liver of each group relative to controls. *$P < 0.05$ and ***$P < 0.001$.

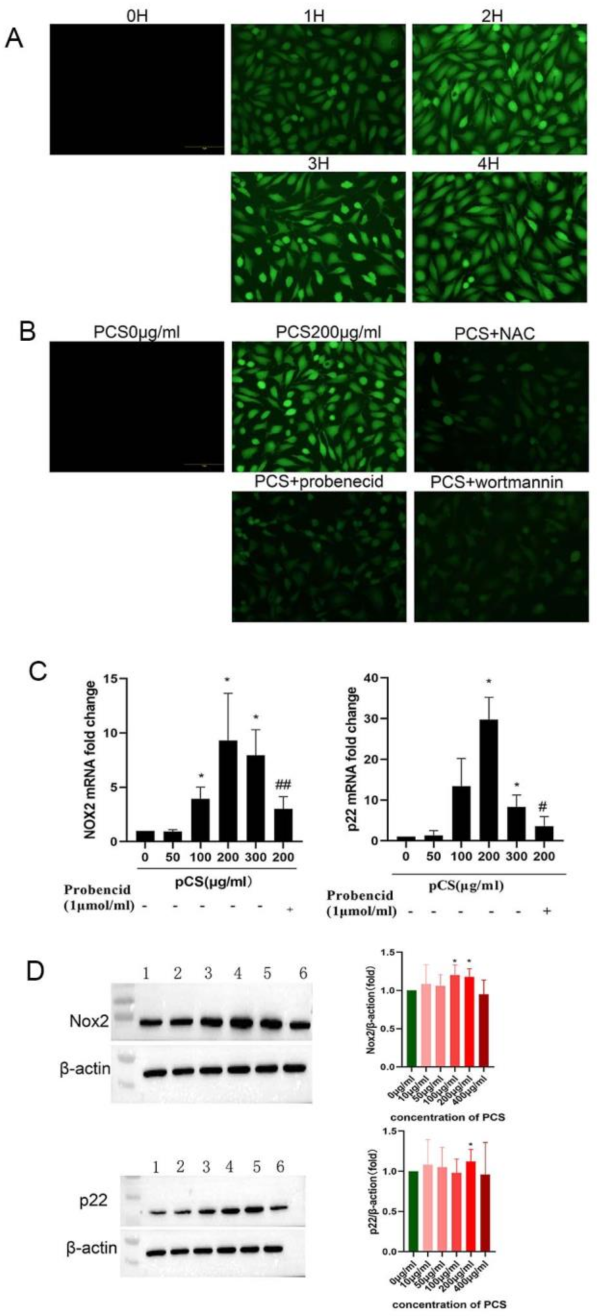

Figure S2. The effect of PCS on bile duct epithelial cells in vitro. (A) The effect of co-culture time on ROS production by bile duct epithelial cells. (B) The effect of different ROS inhibitors on cultured bile duct epithelial cells. (C,D) PCS concentration alters both (C) mRNA and (D) protein levels of NOX2 and P22, and probenecid treatment inhibits NOX2 and P22 production (n = 3/group). *P < 0.05, **P < 0.01 and ***P < 0.001; $^{\#}$P < 0.05, $^{\#\#}$P < 0.01 vs. 200 μg/mL. 1: PCS, 0 μg/mL; 2: PCS, 10 μg/mL;

3: PCS, 50 μg/mL; 4: PCS, 100 μg/mL; 5: PCS, 200 μg/mL; and 6: PCS, 400 μg/mL (n = 3/group).

Supplementary table S1 Composition of tyrosine feed

Ingredient	Tyrosine-rich diet (%)
Casein	20.00
L-Tyrosine	5.00
Cellulose powder	5.00
L-Cystine	0.30
Soybean oil	7.00
AIN-93G mineral mixture	3.50
AIN-93G vitamin mixture	1.00
Choline bitartrate	0.25
Sucrose	10.00
Cornstarch	47.95
Total	100

Supplementary table S2 Primer sequence

Primer		base sequence 5'to 3'	length	Tm value (℃)
IL-6	Forward	TTGCCTTCTTGGGACTGATGT	21	59.5
	Reverse	ATACTGGTCTGTTGTGGGTGGT	22	58.2
TNF-α	Forward	GCCACCACGCTCTTCTGTC	19	58.4
	Reverse	GCTACGGGCTTGTCACTCG	19	58 .9
IL-10	Forward	CAGTCAGCCAGACCCACAT	19	55.7
	Reverse	GGCAACCCAAGTAACCCT	18	54.5
IL-1β	Forward	TACTTGCCGCACGTCCTACAC	21	61.1
	Reverse	ATTTCCGACCCATTCCACTTC	21	60.4
CCL3	Forward	ACTGCCTGCTGCTTCTCCTATG	22	62.3
	Reverse	GCAAAGGCTGCTGGTCTCAAA	21	61.8
Arg-1	Forward	CAAGACAGGGCTACTTTCAG	20	52.5
	Reverse	GATTACCTTCCCGTTTCGTT	20	56.1
NADPH	Forward	CCTTTGTGCCTATACTGTGCT	21	55.1
	Reverse	GTAAATCTCGGAATCTTTCTGTC	20	54.8
TGF-β1	Forward	ATTCCTGGCGTTACCTTG	18	53.1
	Reverse	CCCTGTATTCCGTCTCCTT	19	53.9
GAPDH	Forward	GGTGCTGAGTATGTCGTGGAG	21	57.8
	Reverse	ACAGTCTTCTGAGTGGCAGTGAT	23	58
TIMP-1	Forward	GCCTCTGGCATCCTCTTGTT	20	59.1
	Reverse	CGTCGAATCCTTTGAGCATCT	21	59.4
P22	Forward	TCATCAATGCCTTCTTCTC	19	50
	Reverse	CGTTATCCTCAATGGGTC	18	50
β-actin	Forward	CACAGCTCTGGTGTGTGACAATGGC	20	54.5
	Reverse	GAGCATCATCACCAGCAAAG	20	56.2
